# The effects of living distantly from peritoneal dialysis units on peritonitis risk, microbiology, treatment and outcomes: a multi-centre registry study

**DOI:** 10.1186/1471-2369-13-41

**Published:** 2012-06-15

**Authors:** Yeoungjee Cho, Sunil V Badve, Carmel M Hawley, Stephen P McDonald, Fiona G Brown, Neil Boudville M, Kathryn J Wiggins, Kym M Bannister, Philip Clayton, David W Johnson

**Affiliations:** 1 Australia and New Zealand Dialysis and Transplant Registry, Adelaide, Australia; 2 Department of Nephrology, University of Queensland at Princess Alexandra Hospital, Brisbane, Australia; 3 Department of Nephrology & Transplantation Services, University of Adelaide at the Queen Elizabeth Hospital, Adelaide, Australia; 4 Department of Nephrology, Monash Medical Center, Melbourne, Australia; 5 School of Medicine and Pharmacology, University of Western Australia, Perth, Australia; 6 Department of Renal Medicine,, Royal Melbourne Hospital,, Melbourne, Australia; 7 Department of Nephrology, Royal Adelaide Hospital, Adelaide, Australia; 8 Department of Renal Medicine, Royal Prince Alfred Hospital, Sydney, Australia; 9 Department of Nephrology, Level 2, ARTS Building, Princess Alexandra Hospital, Ipswich Road, Woolloongabba, Brisbane, Qld, 4102, AUSTRALIA

**Keywords:** Antibiotics, Bacteria, Fungus, Microbiology, Peritoneal Dialysis, Peritonitis, Outcomes, Relapse, Remoteness

## Abstract

**Background:**

The aim of the study was to determine whether distance between residence and peritoneal dialysis (PD) unit influenced peritonitis occurrence, microbiology, treatment and outcomes.

**Methods:**

The study included all patients receiving PD between 1/10/2003 and 31/12/2008, using ANZDATA Registry data.

**Results:**

365 (6%) patients lived ≥100 km from their nearest PD unit (distant group), while 6183 (94%) lived <100 km (local group). Median time to first peritonitis in distant patients (1.34 years, 95% CI 1.07-1.61) was significantly shorter than in local patients (1.68 years, 95% CI 1.59-1.77, p = 0.001), whilst overall peritonitis rates were higher in distant patients (incidence rate ratio 1.32, 95% CI 1.20-1.46). Living ≥100 km away from a PD unit was independently associated with a higher risk of *S. aureus* peritonitis (adjusted odds ratio [OR] 1.64, 95% CI 1.09-2.47). Distant patients with first peritonitis episodes were less likely to be hospitalised (64% vs 73%, p = 0.008) and receive antifungal prophylaxis (4% vs 10%, p = 0.01), but more likely to receive vancomycin-based antibiotic regimens (52% vs 42%, p < 0.001). Using multivariable logistic regression analysis of peritonitis outcomes, distant patients were more likely to be cured with antibiotics alone (OR 1.55, 95% CI 1.03-2.24). All other outcomes were comparable between the two groups.

**Conclusions:**

Living ≥100 km away from a PD unit was associated with increased risk of *S. aureus* peritonitis, modified approaches to peritonitis treatment and peritonitis outcomes that were comparable to, or better than patients living closer to a PD unit. Staphylococcal decolonisation should receive particular consideration in remote living patients.

## Background

Peritonitis is a serious complication of peritoneal dialysis (PD), responsible for significant morbidity, and accounting for 30% of technique failures and 21% of infectious deaths in Australian and New Zealand PD patients [1,2]. Studies of organism-specific peritonitis [3-13] have described the frequency, predictors, treatments and clinical outcomes of these conditions. However, the impact of distance of residence from PD unit on PD peritonitis has received scant attention.

Recent analysis of the ANZDATA registry by Lim *et al.*[14] demonstrated greater all-cause mortality in non-metropolitan PD patients in Australia compared with metropolitan PD patients. The remote indigenous PD patients had shorter time to first peritonitis with greater risk of technique failure. Reduced access to specialised medical services and substandard sanitation in remote indigenous patient dwellings have been proposed as reasons for the observed outcomes. This study however defined remoteness based on the Accessibility and Remoteness Index of Australia (ARIA) used by the Australian Bureau of Statistics rather than actual distance to the nearest PD unit.

Distance to the nearest PD unit may influence PD patient outcome by impacting upon access to medical care, delayed diagnosis, delayed dialysate sample processing (leading to higher rates of culture negative peritonitis), compromised management due to the tyranny of distance and ultimately poorer outcomes. These are of particular importance as PD is usually considered a first-choice treatment for end-stage renal disease (ESRD) for patients living in remote areas to avoid relocation [15]. Given that PD peritonitis is a major cause of PD technique failure, addressing the impact of remote residence on PD outcomes, particularly PD peritonitis, is an imperative issue to be addressed. The aim of the current study was to examine the effect of living distantly (≥100 km) from a PD unit on the risk, microbiology, treatment and/or clinical outcomes of PD-associated peritonitis in all Australian PD patients, as recorded in the ANZDATA registry.

## Results

### Population characteristics

A total of 6610 patients received PD in Australia during the study period (1 October 2003 to 31 December 2008) and were followed for 10470 patient-years (mean follow-up 1.58 years per patient). Their characteristics are depicted in Table 1. In this group, 6213 episodes of peritonitis occurred in 3128 (47%) patients (range 1 to 15 episodes per patient). The overall rate of peritonitis was 0.59 episodes per patient-year of treatment (equivalent to 20.2 patient-months per episode).

**Table 1:** Characteristics of Australian PD patients living within or greater than 100 km away from their nearest PD unit during the study period 2003–2008

**Characteristic**	**≥100 km from PD Unit (n = 365)**	**<100 km from PD Unit (n = 6183)**	**P value**
Age (years)	56.9±16.8	59.1±16.9	0.02
Women	197 (54%)	3444 (56%)	0.5
Racial origin			<0.001
Caucasian	229 (63%)	4728 (76%)	
ATSI	130 (36%)	391 (6%)	
MPI	2 (1%)	157 (3%)	
Asian	3 (1%)	628 (10%)	
Other	1 (0%)	279 (5%)	
BMI (kg/m^2^)	26.4±5.4	26.0±5.4	0.2
eGFR at dialysis start (mL/min/1.73 m^2^)	6.7±3.2	7.4±4.1	0.004
Late referral	105 (29%)	1396 (23%)	0.006
ESRD Cause			0.08
Chronic glomerulonephritis	103 (28%)	1697 (27%)	
Diabetic nephropathy	130 (36%)	1806 (29%)	
Renovascular disease	50 (14%)	836 (14%)	
Polycystic kidneys	17 (5%)	348 (6%)	
Reflux nephropathy	11 (3%)	250 (4%)	
Other	37 (10%)	831 (13%)	
Unknown	17 (5%)	415 (7%)	
Smoking status			0.1
Current	55 (15%)	746 (12%)	
Former	142 (39%)	2295 (37%)	
Never	168 (46%)	3142 (51%)	
Chronic lung disease	66 (18%)	847 (14%)	0.02
Coronary artery disease	14 (39%)	2196 (36%)	0.2
Peripheral vascular disease	96 (26%)	1411 (23%)	0.1
Cerebrovascular disease	54 (15%)	836 (14%)	0.5
Diabetes mellitus	160 (44%)	2349 (38%)	0.03
Peritoneal Transport Status			0.001
High	40 (11%)	610 (10%)	
High average	106 (29%)	2230 (36%)	
Low average	70 (19%)	1369 (22%)	
Low	14 (4%)	271 (4%)	
Unknown/Not specified	135 (37%)	1703 (28%)	
Centre size			<0.001
Small (<7 patients)	2 (1%)	42 (1%)	
Small-medium (7–42 patients)	65 (18%)	358 (6%)	
Medium-large (43–140 patients)	62 (17%)	1382 (22%)	
Large (>140 patients)	236 (65%)	4401 (71%)	

Abbreviations: ATSI, Aboriginal and Torres Strait Islander peoples; BMI, body mass index; eGFR, estimated glomerular filtration rate; MPI, Maori and Pacific Islander peoples.

The distance between patient residence and their nearest PD unit was unable to be determined in 62 (1%) patients. For the remaining patients, 365 (6%) lived ≥100 km from their nearest PD unit (distant group), while 6183 (94%) lived <100 km (local group). Their baseline characteristics are shown in Table 1. Compared with local patients, distant PD patients were significantly more likely to be younger, Aboriginal and Torres Strait Islander peoples, have commenced dialysis at a lower level of estimated glomerular filtration rate (eGFR), have chronic lung disease and diabetes mellitus and not have a baseline peritoneal transport status recorded.

Overall peritonitis rates in the distant and local groups were 0.77 (95% CI 0.70-0.85) and 0.58 (95% CI 0.57-0.60) episodes per patient-year, respectively (incidence rate ratio 1.32, 95% CI 1.20-1.46, p < 0.001). When the analysis was restricted to Caucasian patients, overall peritonitis rates in the distant and local groups were 0.67 (95% CI 0.59-0.76) and 0.59 (95% CI 0.57-0.61) episodes per patient-year, respectively (incidence rate ratio 1.14, 95% CI 1.00-1.30, p = 0.05).

### Peritonitis-free survival

**Figure 1:**
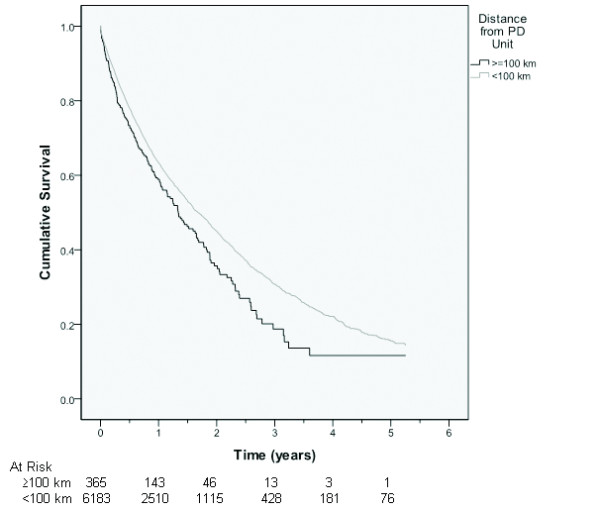
**Kaplan-Meier survival curve for peritonitis-free survival for all Australian patients receiving PD between 1 October 2003 and 31 December 2008, according to whether they lived ≥100 km (n = 365) or <100 km (n = 6183) from their nearest PD unit.** The difference between the 2 Groups was statistically significant (log rank score 10.6, p = 0.001).

Time to first peritonitis episode was significantly shorter in distant patients (Figure 1). Median (95% confidence interval) peritonitis-free survival rates were 1.34 (1.07-1.61) years and 1.68 (1.59-1.77) years, respectively (log rank score 10.6, p = 0.001). Three-year peritonitis-free survival rates were 19% and 31%, respectively. When the analysis was restricted to Caucasians, the 3-year peritonitis-free survival rates were 24% and 31%, respectively.

Using multivariable Cox proportional hazards model analysis, living at least 100 km away from a PD unit was not significantly, independently associated with time to first peritonitis episode (adjusted hazard ratio [HR] 1.11, 95% CI 0.94-1.31). The independent predictors of increased peritonitis hazard were older age, Aboriginal and Torres Strait Islander racial origin, Maori and Pacific Islander racial origin, higher BMI and missing baseline peritoneal transport status. Receiving PD in a small-medium centre (second smallest quartile) was associated with a lower hazard of peritonitis than in the largest quartile.

### Microbiology of first peritonitis episodes

The micro-organisms isolated from dialysate cultures during first episodes of peritonitis in distant and local PD patients during the study period are summarised in Table 2. Compared with local patients, distant patients were significantly more likely to have Gram positive peritonitis due to *Staphylococcus aureus* and *Corynebacteria* and less likely to have non-*Pseudomonas* Gram negative peritonitis. Methicillin-sensitive *S. aureus* accounted for 14% of *S. aureus* isolates in distant patients and 21% of isolates in local patients (p = 0.5). Using multivariable logistic regression, living at least 100 km away from the nearest PD unit was significantly and independently associated with higher odds of *S. aureus* peritonitis (adjusted odds ratio [OR] 1.64, 95% CI 1.09-2.47) and a trend to lower odds of non-*Pseudomonas* Gram negative peritonitis (OR 0.68, 95% CI 0.45-1.01, p = 0.06). Similar results were found when the results were restricted to Caucasian patients (*S. aureus* peritonitis OR 1.60, 95% CI 0.96-2.67, p = 0.07; non-*Pseudomonas* Gram negative peritonitis OR 0.65, 95% CI 0.39-1.08, p = 0.10).

**Table 2:** Micro-organisms isolated from dialysate cultures during first episodes of peritonitis in Australian PD patients during the period 2003–2008, according to patient proximity to their nearest PD unit

**Micro-organism**	**≥100 km from PD Unit (n = 193)**	**<100 km from PD Unit (n = 2915)**	**P value**
Culture-negative	32 (17%)	409 (14%)	0.3
Gram positive	110 (57%)	1555 (53%)	0.3
*S. aureus*	35 (18%)	353 (12%)	0.01
Coagulase-negative staphylococci	51 (26%)	734 (25%)	0.7
Streptococci	19 (10%)	261 (9%)	0.7
Enterococci	8 (4%)	118 (4%)	0.9
Corynebacterium	0 (0%)	58 (2%)	0.05
Pseudomonas	10 (5%)	155 (5%)	0.9
Non-Pseudomonas Gram-negative	33 (17%)	730 (25%)	0.01
Fungal	6 (3%)	116 (4%)	0.5
Mycobacterium	0 (0%)	11 (0.4%)	0.4
Polymicrobial	19 (10%)	362 (12%)	0.3

### Initial empiric antibiotic treatment of first peritonitis episodes

The majority of patients with peritonitis were treated initially with either intraperitoneal vancomycin or cephazolin in combination with gentamicin (Table 3). Compared with local patients, distant patients were significantly more likely to be treated empirically with a vancomycin-based regimen, instead of a cephalosporin-based regimen. Vancomycin-based regimens were also more commonly prescribed in Caucasian patients (51% vs 44%, p = 0.05).

**Table 3:** Initial empiric antibiotic combinations administered to treat first episodes of peritonitis in Australian PD patients during the period 2003–2007, according to patient proximity to their nearest PD unit. The differences between the groups were statistically significant (p < 0.001)

**Antibiotic combination**	**≥100 km from PD Unit (n = 193)**	**<100 km from PD Unit (n = 2915)**
1^st^ generation cephalosporin + aminoglycoside	38 (20%)	1065 (37%)
1^st^ generation cephalosporin + 3^rd^/4^th^ generation cephalosporin	35 (18%)	452 (16%)
First-generation cephalosporin + other Gram-negative antibiotic^a^	4 (2%)	29 (1%)
Vancomycin + aminoglycoside	65 (34%)	974 (33%)
Vancomycin + 3^rd^/4^th^ generation cephalosporin	10 (5%)	55 (2%)
Vancomycin + other Gram-negative antibiotic^a^	25 (13%)	213 (7%)
Other Gram-positive antibiotic^b^ + aminoglycoside	5 (3%)	60 (2%)
Other Gram-positive antibiotic^b^ + 3^rd^/4^th^ generation cephalosporin	1 (11%)	9 (0%)
Other Gram-positive antibiotic^b^ + Other Gram-negative antibiotic^a^	10 (5%)	58 (2%)

^a^Other Gram-negative antibiotics include (in decreasing order of frequency): ciprofloxacin, co-trimoxazole, metronidazole, erythromycin, aztreonam and fleroxacin.

^b^Other Gram-positive antibiotics include (in decreasing order of frequency): flucloxacillin, ampicillin, dicloxacillin, teicoplanin, amoxicillin and erythromycin.

Antifungal chemoprophylaxis was also less commonly co-prescribed in distant patients (4% vs 10%, respectively, p = 0.01), as was heparin (9% vs 23%, p < 0.001). There was no difference between the 2 groups with respect to administration of thrombolytic agents (0% vs 0.3%, p = 0.4). When the analysis was restricted to Caucasian patients, distant patients were less likely to be co-prescribed antifungal prophylaxis (1% vs 4%, p = 0.009) or heparin (5% vs 10%, p = 0.01).

### Outcomes of first peritonitis episodes

Compared with local PD patients, distant patients were significantly less likely to be hospitalised for peritonitis (Table 4). Those distant patients requiring temporary transfer to haemodialysis for peritonitis were significantly more likely to remain on temporary haemodialysis for longer periods of time. Otherwise, peritonitis outcomes between the 2 groups were comparable with respect to cure with antibiotics alone, relapse, catheter removal, permanent haemodialysis transfer and death.

**Table 4:** Clinical outcomes of first episodes of peritonitis in Australian PD patients during the period 2003–2008, according to patient proximity to their nearest PD unit

**Outcome**	**≥100 km from PD Unit (n = 193)**	**<100 km from PD Unit (n = 2915)**	**P value**
Cure with antibiotics	161 (83%)	2283 (78%)	0.1
Relapse	4 (2%)	40 (1%)	0.4
Hospitalisation			
Number (%)	123 (64%)	2115 (73%)	0.008
Duration	6 [3-12]	6 [3-11]	0.8
Catheter removal			
Number (%)	28 (15%)	538 (18%)	0.2
Time to occurrence	7 [2–13.75]	6 [3-12]	0.6
Temporary haemodialysis			
Number (%)	7 (4%)	96 (3%)	0.8
Time to occurrence	5 [2-8]	6.5 [3.25-10]	0.4
Duration	125[88–145]	63.5 [20–94.25]	0.007
Permanent haemodialysis			
Number (%)	22 (11%)	452 (16%)	0.1
Time to occurrence	7.5 [2.75-20.25]	7 [4–12.75]	0.8
Death			
Number (%)	2 (1.0%)	83 (1.8%)	0.4
Time to death	-*	15.5 [1.25-31.25]	0.4

* Unable to provide median [interquartile range] due to occurrence of only 2 deaths in this group.

Results are expressed as number (%) or median days [25^th^ - 75^th^ percentile].

Using multivariable logistic regression analysis, living at least 100 km away from the nearest PD unit was independently predictive of a higher rate of cure with antibiotics alone (OR 1.55, 95% CI 1.03-2.24) and trends to lower rates of catheter removal (OR 0.67, 95% CI 0.43-1.04, p = 0.07) and permanent haemodialysis transfer (OR 0.66, 95% CI 0.40-1.07, p = 0.09). Distance from PD unit was not associated with temporary haemodialysis transfer. The number of events for death and relapse were too small to permit adequate statistical analysis. When the analysis was restricted to Caucasian patients, living at least 100 km away from the nearest PD unit was not independently predictive of cure with antibiotics alone (OR 1.09, 95% CI 0.65-1.83), catheter removal (OR 0.95, 95% CI 0.54-1.66) or permanent haemodialysis transfer (OR 0.86, 95% CI 0.47-1.57).

## Discussion

The present study represents the largest examination to date of the effect of distance from PD unit on the frequency and clinical outcomes of PD-associated peritonitis. Distant group patients were younger, more likely of indigenous origin and treated by a small PD unit. Time to first peritonitis episode was significantly shorter in this distant group and a greater proportion experienced *S. aureus* and *Corynebacteria* peritonitis. Distance from PD unit was independently associated with a higher risk of *S. aureus* peritonitis. Distant patients were more likely to receive a vancomycin-based antibiotic regimen and be cured with antibiotics alone, and tended to be less likely to undergo catheter removal or permanent haemodialysis transfer.

Greater representation of indigenous patients in remote communities initiating PD and shorter times to first peritonitis episodes in indigenous non-metropolitan patients have been previously reported [14,16]. However, these investigations defined remoteness based on the Accessibility and Remoteness Index of Australia (ARIA) used by the Australian Bureau of Statistics. In contrast, the present study defined remoteness as living at least 100 km away from the nearest PD unit, which is arguably better suited to reflect accessibility to specialist medical care. The present study also examined peritonitis risk, microbiology, treatment and outcomes in both indigenous and non-indigenous PD patients.

Though culture-negative PD peritonitis did not differ between the two groups, the distant patients were more likely to experience peritonitis due to *S. aureus* and *Corynebacteria* and less likely to encounter non-Pseudomonas gram-negative (NPGN) PD peritonitis. Following multivariable adjustment, distance from PD unit was independently associated with a higher risk of *S. aureus* peritonitis. The reasons for this finding are uncertain, but may reflect a higher rate of staphylococcal colonisation in distant patients or a reduced probability of initiating staphylococcal decolonisation procedures in this group. Chronic carriage of *S. aureus* has been recognised as being very important in the development of peritonitis in patients on PD since a previous study of Brazilian PD patients found that 95% with both nasal and pericatheter staphylococcal colonization were colonized with the same subtypes at both sites and 100% of patients with *S. aureus* peritonitis were infected with a subtype which colonized the nares, pericatheter skin or both [17]. A recent meta-analysis of 13 studies (including 3 randomized controlled trials) demonstrated that topical mupirocin application to the nares or exit sites of PD patients reduced the risk of *S. aureus* exit site infection by 72% (95% CI 0.60-0.81) and peritonitis by 70% (95% CI 0.52-0.81) [18]. Although the ANZDATA Registry does not collect information about topical nasal and/or exit site anti-staphylococcal prophylaxis, it is conceivable that distant patients were less likely to receive such prophylaxis, particularly since the present study demonstrated that patients living more than 100 km away from their nearest PD unit were significantly less likely to receive anti-fungal chemoprophylaxis. A previously published prospective survey of contemporary infection prophylaxis practice in Australia and New Zealand PD units demonstrated poor adherence to national and international best practice guidelines and absence of a uniform, standard practice of exit site care. In particular, 47% had no fixed exit site infection prophylaxis policy, 53% did not routinely prescribe exit site or nasal mupirocin, 57% did not screen for nasal *S. aureus* and 92% did not co-prescribe anti-fungal prophylaxis [19,20]. It remains unknown whether this variable adherence to guidelines is influenced by the distance of patient residence from the PD unit.

A vancomycin-based regimen was more commonly used to treat peritonitis in distant patients, than those living within 100 km of their PD unit. This therapeutic decision may have been influenced by the lower probability of hospitalisation of remote patients (probably reflecting logistic considerations) and the convenience of a less frequent dosing requirement for vancomycin, which was more suited to outpatient treatment. Clinical outcomes with vancomycin-based regimens were comparable to those of cephalosporin-based regimens and superior to those of other Gram positive agents.

Indeed, the overall clinical outcomes of peritonitis episodes in patients living at least 100 km away from the nearest PD unit were comparable with those of patients residing closer to nephrologic care. When adjustments were made for differences in baseline characteristics (including an over-representation of Aboriginal and Torres Strait Islanders in remote living patients), distant patients achieved higher cures rates with antimicrobial agents alone and lower rates of catheter removal or permanent haemodialysis transfer. These findings may reflect a stronger incentive to persist with antibiotics in remote living patients rather than removing the PD catheter and transferring to haemodialysis since such an action might have significant social implications for the patient, such as relocation closer to the dialysis service and dislocation from their family and community. Nevertheless, the time to catheter removal did not differ significantly between distant and local patients.

These findings contrast somewhat with a previously published Canadian registry study [15], which found that living remotely from a PD unit was associated with increased risks of PD technique failure and mortality. As with the present study, remote patients were more likely to be younger, Aboriginal and have diabetes mellitus and chronic lung disease. The causes of technique failure and death were not reported in the Canadian study, so it is conceivable that these adverse clinical outcomes could be related to factors other than peritonitis. Moreover, in Canada, 6% of PD patients lived more than 300 km away from their nearest PD unit, such that it is possible that comorbidities may not have been as well captured in these extremely remote living patients. It is also possible that other factors may have contributed to an apparent disparity in the findings between the two studies, such as the local availability of primary healthcare, pathology and telemedicine services in remote regions. On the other hand, another Canadian study found no association relationship between distance of patient residence (≤50 km or > 50 km from the primary dialysis centre in Winnipeg) and peritonitis-free, technique or patient survival rates among patients who were Aboriginal [21]. The apparent disparity in results may relate to the smaller sample size of the Manitoba study (n = 727).

Recently, the BRAZPD study [22] reported paradoxically higher peritonitis rates in patients living within 25 km of a PD unit compared with those living more than 50 km away from the unit (HR 1.40, 95% CI 1.07-1.83). They speculated that these results may have reflected poorer hygiene conditions in urban settings compared with more distant sites. In contrast, urban conditions in Australia are likely to be quite different to those in Brazil. The BRAZPD study also found that larger PD units were associated with a smaller risk of peritonitis [22], whilst our study observed that receiving PD in a small-medium centre (second smallest quartile) was associated with a lower hazard of peritonitis than in the largest quartile. In contrast, no association was observed between PD centre size and peritonitis risk in London [23] or Scotland [24]. Whilst increasing centre size may lead to increased PD experience and improved peritonitis prevention, it is also conceivable that excessively large centres may be operationally inefficient leading to suboptimal infection control procedures and increased peritonitis risks. Alternatively, the results of studies to date may have been limited by residual confounding due to a failure to adjust for other facility variables that contribute to “centre effects.”

The strengths of this study include its very large sample size and inclusiveness. We included all patients receiving PD in Australia during the study period, such that a variety of centres were included with varying approaches to the microbiological diagnosis and treatment of peritonitis. This greatly enhanced the external validity of our findings. These strengths should be balanced against the study’s limitations, which included limited depth of data collection. ANZDATA does not collect important information, such as the presence of concomitant exit site and tunnel infections, antimicrobial susceptibilities of isolated micro-organisms, patient compliance, socio-economic status, individual unit management protocols (including the approaches to staphylococcal decolonisation), duration of cloudy dialysate, laboratory values (such as C-reactive protein and dialysate white cell counts), severity of comorbidities, PD modality (ambulatory or continuous ambulatory PD) at time of peritonitis, disconnect systems used, prescribed PD dialysate (especially icodextrin), antibiotic dosages or routes of antibiotic administration, peritoneal dialysate culture methodology, previous antibiotic exposure for any indication, or local availability of health services in different remote regions (eg nephrologist outreach, primary healthcare, pathology services, telemedicine, etc.). Even though we adjusted for a large number of patient characteristics, the possibility of residual confounding could not be ruled out. Moreover, the relatively small number of patients in the distant group (n = 193) may have reduced the power of the study to identify other independent predictors of peritonitis risk. Patients without a valid postcode were excluded, although this group only accounted for 1% of all PD patients. Distance from PD unit was based on the patient’s residence at the commencement of PD. Thus, it is possible that some patients moved from < to ≥ 100 km away from the PD unit or vice versa during their PD career, thereby leading to misclassification bias. In common with other Registries, ANZDATA is a voluntary Registry and there is no external audit of data accuracy, including the diagnosis of peritonitis. Consequently, the possibility of coding/classification bias cannot be excluded.

## Conclusion

Our study represents the largest examination to date of the effect of living distantly from a PD unit on the risk, microbiology, treatment and clinical outcomes of PD-associated peritonitis. Living at least 100 km away from a PD unit was independently associated with a heightened risk of *S. aureus* peritonitis, modified approaches to peritonitis treatment (including less frequent hospitalisation, greater reliance on vancomycin-based regimens and a tendency to persist with antimicrobial agents in preference to catheter removal) and superior antimicrobial cure rates compared with patients living within 100 km of a PD unit. The heightened risk of *S. aureus* peritonitis and lower use of antifungal prophylaxis in remote living PD patients highlights the need to focus on implementation of evidence-based infection control strategies (such as staphylococcal decolonisation) in this patient group. Further research evaluating the effectiveness of different guideline implementation strategies on peritonitis prevention, particularly in remote-living PD patients, is warranted.

## Methods

### Study population

The study included all Australian adult patients from the ANZDATA Registry who were receiving PD between 1^st^ October 2003 and 31^st^ December 2008. Collection and analysis of ANZDATA Registry data was approved by the Princess Alexandra Hospital Human Research Ethics Committee. Permission to analyse the data was also granted by the ANZDATA Registry Executive. The data collected included demographic data, cause of primary renal disease, co-morbidities at the start of dialysis, smoking status, body mass index (BMI), late referral (defined as commencement of dialysis within 3 months of referral to a nephrologist), microbiology of peritonitis episodes (up to three organisms for polymicrobial episodes) and the initial and subsequent antibiotic treatment regimens. Centre size was categorised according to quartiles of the numbers of patients cared for by individual units over the duration of the study: small (<7 patients), small-medium (7–42 patients), medium-large (43 – 140 patients) and large (>140 patients).

Patients were analysed according to whether their residence was located ≥100 km (distant) or <100 km (local) from their nearest PD unit. Distance between patient residence (based on postcode) and peritoneal dialysis unit was determined using Google maps (http://www.maps.google.com.au). This distance represented travelling distance, rather than straight line distance. The distance of 100 km was chosen *a priori* because this is the minimum distance for which most state governments of Australia provide patient-assisted transport subsidy schemes to facilitate improved access of remote living patients to specialised medical care.

Peritonitis was defined as clinical features of peritonitis (abdominal pain or cloudy dialysate) and dialysate leukocytosis (white blood cell count > 100/μL with > 50% neutrophils). Peritonitis rates were calculated according to the standardised recommendations made by the International Society of Peritoneal Dialysis (ISPD) [25,26].

The outcomes examined were peritonitis rate, peritonitis-free survival, microbiology, antimicrobial therapy, cure, relapse, peritonitis-associated hospitalization, catheter removal, temporary haemodialysis transfer (in which patients subsequently resumed PD), permanent haemodialysis transfer and patient death. A peritonitis episode was considered “cured” by antibiotics alone if the patient was symptom free, the PD effluent was clear and the episode was not complicated by relapse, catheter removal or death. Peritonitis-related death was recorded if the patient’s death was directly attributable to peritonitis in the clinical opinion of the treating nephrologist. In view of the complexities associated with analysis of multiple events within individuals where the assumption of independence of observations is not appropriate, only the first episodes of peritonitis from each individual were included when analysing all outcomes, except peritonitis rates.

### Statistical analysis

Results were expressed as frequencies and percentages, mean ± standard deviation or median [25^th^ - 75^th^ percentile], as appropriate. Differences between groups were analysed by chi-square test for categorical data, unpaired *t*-test for continuous normally distributed data and Mann–Whitney test for continuous non-normally distributed data. Peritonitis rates were compared by Poisson regression analysis. Peritonitis-free survival was determined by Kaplan-Meier survival analysis and by multivariable, Cox proportional hazards model analysis using backward stepwise elimination. The independent predictors of the clinical outcomes of peritonitis were determined by multivariable logistic regression. Distance from PD unit, age, gender, racial origin, BMI, eGFR at dialysis start, late referral within 3 months of dialysis commencement, ESRD cause, smoking status, comorbidities, peritoneal transport status, climatic region, centre size, isolated organism, initial empiric antibiotic regimen and isolated micro-organisms were included in the model as covariates. Assumptions for the Poisson regression, logistic regression and Cox proportional hazards models were verified. First-order interaction terms between the significant covariates were examined, where appropriate. Data were analysed using the software package PASW Statistics for Windows release 18.0 (SPSS Inc., North Sydney, Australia). P values less than 0.05 were considered statistically significant.

## Abbreviations

ANZDATA, Australian and New Zealand Dialysis and Transplant Registry; ARIA, Accessibility and Remoteness Index of Australia; ATSI, Aboriginal or Torres Strait Islander; BMI, Body mass index; eGFR, Estimated glomerular filtration rate; ESRD, End-stage renal disease; HR, Adjusted hazard ratio; ISPD, International Society of Peritoneal Dialysis; MPI, Maori or Pacific Islander; NPGN, Non-pseudomonas Gram-negative organism; OR, Adjusted odds ratio; PD, Peritoneal dialysis.

## Competing interests

Professor David Johnson is a consultant for Baxter Healthcare Pty Ltd and has previously received research funds from this company. He has also received speakers’ honoraria and research grants from Fresenius Medical Care and has previously been a consultant to Gambro. He is supported by a Queensland Government Health Research Fellowship. Dr Kym Bannister is a consultant for Baxter Healthcare Pty Ltd. Dr Fiona Brown is a consultant for Baxter and Fresenius and has received travel grants from Amgen and Roche. Dr Stephen McDonald has received speaking honoraria from AMGEN Australia, Fresenius Australia and Solvay Pharmaceuticals and travel grants from AMGEN Australia, Genzyme Australia and Jansen-Cilag. Associate Professor Neil Boudville has previously received research funds from Roche, travel grants from Roche, Amgen and Jansen Cilag, and speaking honoraria from Roche. The remaining authors have no competing financial interests to declare.

## Authors’ contributions

YC and DJ conceived of the study; participated in the study design and the analysis and interpretation of the results; helped to draft the manuscript. SB, CH, SM, FB, NB, KW, KB and PC participated in the study design and the analysis and interpretation of the results. All authors read and approved the final manuscript.

## Pre-publication history

The pre-publication history for this paper can be accessed here:

http://www.biomedcentral.com/1471-2369/13/41/prepub
